# Predictive value of HMGB1 for atrial fibrillation recurrence after cryoballoon ablation in paroxysmal atrial fibrillation patients

**DOI:** 10.1002/clc.23904

**Published:** 2022-09-20

**Authors:** Xinxin Li, Cuiting Zhao, Meng Li, Hongxiao Yu, Xiping Liu, Qing Zhu, Xiaokun Song, Yonghuai Wang, Bo Yu, Chunyan Ma

**Affiliations:** ^1^ Department of Cardiovascular Ultrasound The First Hospital of China Medical University Shenyang Liaoning China; ^2^ Department of Cardiology The First Hospital of China Medical University Shenyang Liaoning China

**Keywords:** atrial fibrillation recurrence, cryoballoon ablation, HMGB1, paroxysmal atrial fibrillation

## Abstract

**Background:**

Cryoballoon ablation (CBA) is recommended for patients with symptomatic drug refractory paroxysmal atrial fibrillation (pAF). However, substantial atrial fibrillation (AF) recurrence is common during follow‐up. Searching for a potential biomarker representing both myocardial injury and inflammation to identify patients at high risk of AF recurrence after CBA is very meaningful for postoperative management of AF patients.

**Hypothesis:**

To evaluate the clinical efficacy of high‐mobility group box 1 (HMGB1) protein released from the left atrium to predict AF recurrence in pAF patients after CBA at 1‐year follow‐up.

**Methods:**

We included 72 pAF patients who underwent CBA. To determine the expression levels of HMGB1, left atrial blood samples were collected from the patients before CBA and after the procedure through the transseptal sheath. Patients were followed up for AF recurrence for 1 year.

**Results:**

A total of 19 patients of the 72 experienced AF recurrence. The level of postoperative HMGB1 (HMGB1post) was higher in the AF recurrence group than in the AF non recurrence group (*p* = .03). However, no differences were noted in the levels of other biomarkers such as preoperative high‐sensitivity C‐reactive protein (hs‐CRP), postoperativehs‐CRP, and preoperative HMGB1 between the two groups. Multiple logistic regression analysis revealed that a higher level of serum HMGB1post was associated with AF recurrence (odds ratio: 5.29 [1.17–23.92], *p* = .04). Receiver operating characteristic analysis revealed that HMGB1post had a moderate predictive power for AF recurrence (area under the curve: 0.68; sensitivity: 72%; and specificity: 68%). The 1‐year AF‐free survival was significantly lower in patients with a high HMGB1post level than in those with a low HMGB1post level (hazard ratio: 3.81 [1.49–9.75], *p* = .005).

**Conclusion:**

In pAF patients who under went CBA, the level of HMGB1 after CBA was associated with AF recurrence and demonstrated a moderate predictive power. Thus, we offer a potential biomarker to identify pAF patients at high risk of AF recurrence.

## 1 INTRODUCTION

Atrial fibrillation (AF), a frequent type of supraventricular arrhythmia, is the most common atrial arrhythmia among adults worldwide, and its prevalence has been increasing.^1^ AF is a progressive disease and more than 50% of the paroxysmal atrial fibrillation (pAF) patients progress to persistent AF or die within 10 years.^2^ Thus, early intervention in pAF is of great significance.

Currently, catheter ablation (CA) is recommended for patients with symptomatic drug refractory pAF.^3^, ^4^ However, substantial AF recurrence is common during follow‐up, which overshadows the potential benefits of the procedure.^5^ As CA techniques for AF have significantly evolved, cryoballoon ablation (CBA) was found to have significantly fewer repeat ablations than radiofrequency ablation,^6^ and was superior to drug therapy for the prevention of atrial arrhythmia recurrence in patients with pAF.^7^ However, there is still a recurrence rate of about 20%–30% in the follow‐up 1 year after CBA.^8^ CBA processes can trigger myocardial injury and inflammatory response,^9^ and inflammation were reported to play important roles in AF recurrence.^10^, ^11^ But the precise pathways of inflammation at AF are still unclear, it is assumed that inflammatory cytokines can bind to atrial myocytes inducing tissue damage.^12^ In line with this hypothesis, searching for a potential biomarker representing both myocardial injury and inflammation to identify patients at high risk of AF recurrence within 1 year after CBA is very meaningful for postoperative management of AF patients.

High‐mobility group box 1 (HMGB1) protein is a secreted protein that can be released into the extracellular space by damaged cells during injury. In addition, the release of HMGB1 by necrotic cells can trigger inflammation.^13^ The Previous study found that the expression levels of HMGB1 were significantly higher in AF patients and in the left atrium than in normal patients and in the periphery, respectively.^14^ A review concluded that HMGB1 may be released by the left atrium, and it may mediate inflammatory processes after CBA and influence the occurrence and development of AF.^15^

Although these studies have presented the association between HMGB1 and AF, the possibility of HMGB1 released from the left atrium predicting AF recurrence remains unclear. Therefore, this study was designed to investigate the predictive value of serum HMGB1 released from the left atrium for AF recurrence.

## 2 METHODS

### 2.1 Study population

Study subjects were consecutively included between August 2017 and August 2018 in the cardiology department at the First Hospital of the China Medical University. All patients were older than 18 years, suitable for CBA, and had voluntarily participated in the study. AF was diagnosed with a 12‐lead electrocardiogram (ECG) or Holter ECG. The diagnostic standard was in accordance with the pAF definition of the American Heart Association.^16^ We excluded pAF patients who had severe heart failure, severe valvular disease, left atrial (LA) thrombosis, coagulation abnormalities, malignancies, thyroid dysfunction, and hepatic or renal dysfunction that were not suitable for ablation. In addition, pAF patients with autoimmune, inflammatory diseases, and a 6‐month surgical history were excluded, because these factors may affect their HMGB1 levels. Written informed consent was obtained from all patients before enrollment, and the study complied with the Declaration of Helsinki.

### 2.2 Echocardiographic measurements

All patients underwent transthoracic echocardiography within 72 h before CBA. Transthoracic echocardiography was performed using the Vivid E9 ultrasound system (GE Healthcare). The diameter of the left atrium was measured in the parasternal long‐axis view on the two‐dimensional image. Simpson's biplane method was used to measure left ventricular ejection fraction (LVEF) as well as LA volume in the apical four‐chamber and two‐chamber views. Notably, the LA appendage should not be included in the tracing of the endocardial border. Left atrial volume index (LAVI) was calculated by dividing the estimated end‐systolic LA volume by the body surface area. All measurements were performed in accordance with the American Society of Echocardiography guidelines.^17^

### 2.3 Ablation protocol

Transesophageal echocardiography was used to exclude any left atrium or LA appendage thrombosis. Multidetector computed tomography of pulmonary vein (PV) was performed 24 h before CBA to assess PV anatomy. Antiarrhythmic drugs were discontinued at least five half‐lives before CBA and anticoagulation drugs were administered during and after CBA. The ablation mode was unified, in brief, a second‐generation 28‐mm cryoballoon (Medtronic) was delivered using a transseptal puncture and an over‐the‐wire delivery technique. The balloon was placed at the ostium of each PV, and repeated cryoapplications were conducted until blocking tests confirmed PV isolation. When the temperature fell below −56°C, the freezing was stopped immediately to prevent largely irreversible damage to the myocardium. None of the patients performed a bonus freeze. Further details about the CBA procedure can be found in the international expert consensus.^18^ The ablation was considered successful if all tested pulmonary potentials with a mapping catheter were completely blocked between the atrial tissue and PV.

### 2.4 Blood sample collection and postprocessing

Transseptal puncture was performed using a transseptal sheath during CBA. After access to the left atrium was achieved using the sheath, blood samples for determining the preoperative HMGB1 (HMGB1pre) level were obtained before the formal procedures of CBA. Similarly, after successful CBA, blood samples were immediately collected through the sheath and placed in the left atrium for determining the postoperative HMGB1 (HMGB1post) level. Thereafter, the blood samples were centrifuged at 3000 rpm for 10 min, and the serum samples were stored at −80°C until analysis. The inflammatory biomarkers, high‐sensitivity C‐reactive protein (hs‐CRP) and HMGB1, were measured using commercial Enzyme‐linked Immunosorbent Assay (Boster Biological Technology) Kits according to the manufacturer's instructions.

### 2.5 Follow‐up

After CBA, all patients underwent continuous electrocardiographic monitoring until discharge and were prescribed oral anticoagulation for at least 3 months based on the CHA2DS2‐VASc score. The endpoint was AF recurrence, defined as episodes of AF or atrial tachyarrhythmia lasting more than 30 s diagnosed with a 12‐lead ECG or Holter ECG, after a 90‐day blanking period.^16^ Patients' follow‐up was conducted at 3, 6, and 12 months after the ablation procedure and whenever required due to symptoms of AF, through outpatient clinic visits or contacting by phone from patients, their family members and referring physicians.

### 2.6 Statistical analysis

The baseline data were stratified by pAF recurrence after CBA. Continuous data are expressed as means ± standard deviation or medians (interquartile range), and categorical data are expressed as counts and percentages. Intergroup comparisons were performed using unpaired Student *t*‐tests for normally distributed variables and the nonparametric Mann–Whitney *U* test for nonnormally distributed variables. Categorical variables were collated using the *χ* ^2^ test or Fisher's exact test. Multivariate regression analysis was performed to analyze the risk factors for the prediction of AF recurrence. The results are expressed as odds ratios (ORs) and 95% confidence intervals. Optimal cutoff values were determined with Youden's index by analyzing the sensitivity and specificity values derived from the receiver operating characteristic (ROC) curve data. The area under the curve (AUC) was calculated to assess the discriminatory power of HMGB1post. The AF‐free survival rates were determined using Kaplan–Meier curves and compared using the logrank test. Data were analyzed using SPSS ver. 25 (IBM SPSS Statistics for Windows, version 25.0). The results of the *t*‐test and *χ* ^2^ test were validated by bootstrapping (1000 repetitions). *p* < .05 was considered significant.

## 3 RESULTS

### 3.1 Study population

The study flowchart is shown in Supporting Information: Figure 1. A total of 72 patients were enrolled in the study. Nineteen (26%) of the 72 pAF patients experienced AF recurrence, whereas 53 (74%) patients did not experience postoperative recurrence. Characteristics of patients were stratified by recurrence and nonrecurrence of AF. Age, sex, AF duration, CHADS‐VASC score, concomitant diseases, biochemical index, and echocardiographic parameters (left atrial diameter, LVEF, and LAVI) were not significantly different between the AF recurrence and AF nonrecurrence groups. The results were consistent after bootstrapping (1000 times; Table 1).

**Table 1:** Comparison of clinical and basic information of paroxysmal atrial fibrillation (AF) patients of the two groups

	All patients	Nonrecurrence	Recurrence	*p*‐Value after bootstrapping
	(*N* = 72)	(*N* = 53)	(*N* = 19)	
*Clinical characteristics*				
Age (years)	61.5 ± 8.8	61.6 ± 8.9	61.2 ± 8.8	.84
Female (*n* [%])	21 (29%)	16 (30%)	5 (26%)	1.00
AF period (months)	61.5 ± 67.5	60.3 ± 62.6	64.9 ± 81.2	.95
CHA2DS2‐VASc score	2.00 ± 1.50	1.98 ± 1.52	1.76 ± 1.25	.31
Hypertension (*n* [%])	32 (48%)	27 (51%)	5 (26%)	.11
Diabetes mellites (*n* [%])	10 (14%)	8 (15%)	2 (11%)	1.00
Peripheral arterial disease (*n* [%])	14 (20%)	11 (21%)	3 (16%)	.75
Fasting blood glucose (mmol/L)	5.41 ± 1.48	5.40 ± 0.96	5.44 ± 2.45	.92
Total cholesterol (mmol/L)	3.74 ± 1.01	3.66 ± 1.02	3.97 ± 1.00	.26
LDL‐cholesterol (mmol/L)	2.15 ± 1.02	2.23 ± 1.04	2.07 ± 0.98	.92
HDL‐cholesterol (mmol/L)	2.42 ± 1.73	2.33 ± 1.72	2.40 ± 1.68	.67
Triglycerides (mmol/L)	1.62 ± 0.83	1.56 ± 0.68	1.84 ± 1.19	.64
Uric acid (μmol/L)	296.22 ± 83.71	302.25 ± 85.69	279.11 ± 77.53	.32
Creatinine (μmol/L)	67.71 ± 13.15	67.22 ± 12.87	65.28 ± 14.18	.59
*Echocardiographic parameters*				
Left atrial diameter (mm)	39.65 ± 5.06	40.15 ± 4.90	38.48 ± 5.70	.24
Left atrial volume index (ml/m^2^)	33.42 ± 10.18	34.19 ± 10.49	31.04 ± 9.04	.56
Left ventricular ejection fraction (%)	62.70 ± 4.37	63.23 ± 4.59	61.65 ± 3.62	.15

*Note*: The values shown are mean ± SD or percentages.

Abbreviations: HDL, high‐density lipoprotein; LDL, low‐density lipoprotein.

In 72 patients, the number of PVs totaled 288. The mean nadir temperature for the left inferior pulmonary vein (LIPV), left superior pulmonary vein (LSPV), right inferior pulmonary vein (RIPV), and right superior pulmonary vein (RSPV) were recorded for all cryoablations, as shown in Table 2. The results revealed that there were no significant differences between AF recurrence and AF nonrecurrence group for nadir temperature of all veins. PV isolation was achieved with the CBA in 100% veins. And the number of applications achieving PV isolation did not differ between the two groups in the veins (LSPV: 1.3 ± 0.6 vs. 1.2 ± 0.5, *p* = .56; LIPV: 1.3 ± 0.5 vs. 1.4 ± 0.6, *p* = .54; RSPV: 1.3 ± 0.5 vs. 1.3 ± 0.7, *p* = .72; RIPV: 1.3 ± 0.6 vs. 1.2 ± 0.4; *p* = .38; Supporting Information: Table 1).

**Table 2:** Comparison of high‐sensitivity C‐reactive protein and high‐mobility group box 1 (HMGB1) protein from the left atrial serum between the two groups

	All subjects	Nonrecurrence	Recurrence	*p*‐Value after bootstrapping
	(*N* = 72)	(*N* = 53)	(*N* = 19)	
Preoperation				
hs‐CRPpre (mg/L)	4.47 (3.79–5.15)	4.36 (3.74–5.08)	4.80 (3.74–8.03)	.71
HMGB1pre (μg/L)	221.73 (191.18–250.50)	221.15 (193.35–246.98)	239.15 (189.63–360.70)	.37
Postoperation				
hs‐CRPpost (mg/L)	41.96 (36.48–50.13)*	43.17 (36.10–50.24)*	40.39 (36.97–69.60)*	.63
HMGB1post (μg/L)	268.78 (244.24–298.79)*	260.28 (239.14–295.39)*	289.73 (270.29–365.42)*	.03

*Note*: The values shown are percentiles.

Abbreviations: HMGB1post, postoperative HMGB1; HMGB1pre, preoperative HMGB1; hs‐CRPpost, postoperative high‐sensitivity C‐reactive protein; hs‐CRPpre, preoperative hs‐CRP.

* *p* < .05 versus pre‐operation

### 3.2 Laboratory test

The results revealed that no obvious differences existed between the two groups for the preoperative high‐sensitivity C‐reactive protein (hs‐CRPpre) and HMGB1pre levels. The levels of hs‐CRP and HMGB1 in the left atrium were significantly elevated after ablation. Moreover, the HMGB1post levels were obviously higher in the AF recurrence group than in the AF nonrecurrence group (289.73 vs. 260.28 µg/L; *p* = .03). However, the postoperative hs‐CRP (hs‐CRPpost) levels did differ between the two groups (Table 2).

**Figure 1:**
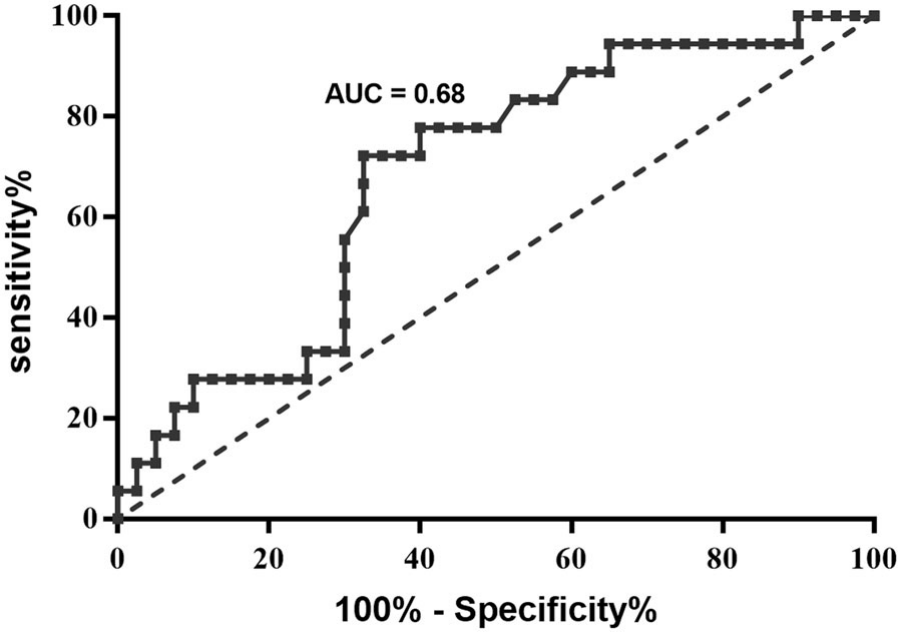
Receiver‐operating characteristic analysis of postoperative high‐mobility group box 1. AUC, area under the curve.

**Figure 2:**
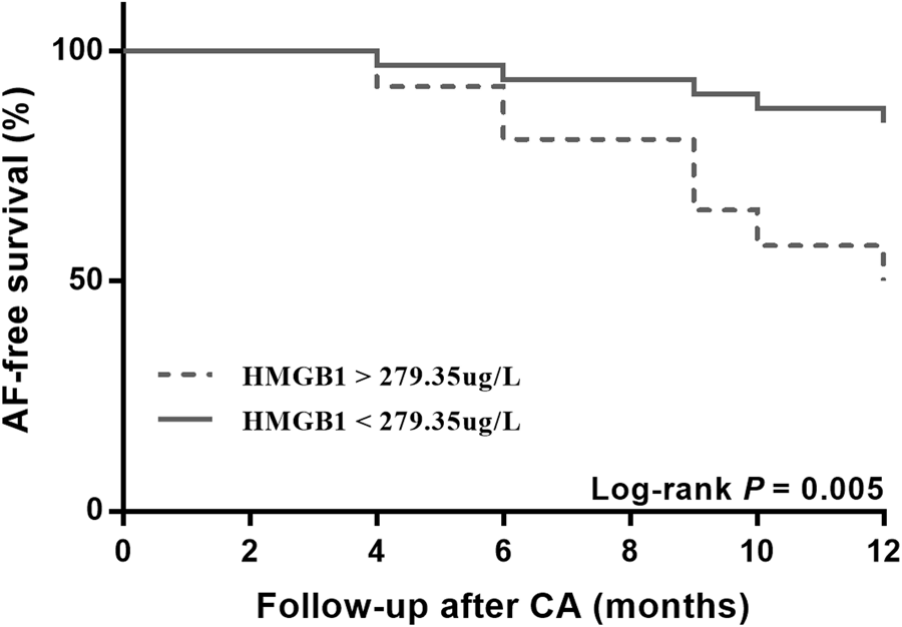
Kaplan–Meier survival curve for atrial fibrillation (AF) reoccurrence. Analysis of 1‐year AF‐free survival to determine the impact of postoperative high‐mobility group box 1 (HMGB1) protein on AF recurrence in paroxysmal AF patients after catheter ablation (CA).

Univariate logistic regression analysis revealed that serum HMGB1post released from the left atrium was associated with AF recurrence (OR: 3.90 [1.16–13.08], *p* = .03). Next, some common clinical risk factors for AF recurrence were adjusted in a multivariate logistic regression analysis based on previous literature,^19^, ^20^, ^21^ clinical knowledge, and statistically significant covariates in univariate analysis. The risk factors included age, female sex, AF period, CHADS2‐VASc score, hypertension, diabetes mellitus, peripheral arterial diseases, LA diameter, LAVI, LVEF, and HMGB1post. The multiple logistic regression analysis confirmed that a high LA serum HMGB1post level had a significant association with AF recurrence (OR: 3.62 [1.02–12.77], *p* = .04; Table 3). ROC analysis revealed that serum HMGB1post released from the left atrium (AUC: 0.68; sensitivity: 72%; specificity: 68%) had a moderate predictive power for AF recurrence and the cutoff value of serum HMGB1post was calculated to be 279.35 µg/L (Figure 1). Kaplan–Meier survival curves for AF recurrence using the HMGB1post values revealed that the 1‐year AF‐free survival was significantly lower in patients with a high serum HMGB1post level (HMGB1post: ≥ 279.35 µg/L) than in those with a low HMGB1post level (HMGB1post: < 279.35 µg/L) (hazard ratio: 3.81 [1.49–9.75], *p* = .005; Figure 2).

**Table 3:** Prediction of paroxysmal atrial fibrillation (AF) recurrence

	Univariate		Multivariate	
	OR (95% CI)	*p*‐Value	OR (95% CI)	*p*‐Value
Age (years)	1.00 (0.94–1.06)	.87	0.96 (0.88–1.06)	.45
Female (%)	0.83 (0.25–2.68)	.75	1.18 (0.26–5.46)	.83
AF period (months)	1.00 (0.99–1.01)	.80	1.00 (0.99–1.01)	.64
CHA2DS2‐VASc score	0.86 (0.60–1.23)	.41	0.91 (0.44–1.87)	.79
Hypertension (%)	0.34 (0.11–1.09)	.07	0.71 (0.17–2.99)	.64
Diabetes mellitus (%)	0.66 (0.13–3.44)	.62	1.65 (0.19–14.43)	.65
Peripheral arterial diseases (%)	0.72 (0.18–2.90)	.64	1.64 (0.19–14.84)	.66
Left atrial diameter (mm)	0.93 (0.84–1.04)	.23	0.90 (0.76–1.07)	.22
Left atrial volume index (ml/m^2^)	0.98 (0.93–1.04)	.48	1.02 (0.93–1.11)	.69
Left ventricular ejection fraction (%)	0.91 (0.80–1.04)	.15	0.88 (0.74–1.09)	.27
HMGB1post (μg/L)	3.90 (1.16–13.08)	**.03**	5.29 (1.17–23.92)	**.04**

*Note*: Bold value means *p* < .05.

Abbreviations: CI, confidence interval; HMGB1post, postoperative high‐mobility group 1 protein; OR, odds ratio.

## 4 DISCUSSION

Recently, CBA has been found to be superior to antiarrhythmic drugs for preventing AF recurrence and improving AF‐specific quality of life and symptoms when used as an initial first‐line rhythm control strategy in pAF patients.^7^, ^22^, ^23^, ^24^ By inducing frozen damage on the myocardial cells, CBA forms a conduction block at the ablation site. However, this process causes an injury to the atrium tissue and inflammation.^25^, ^26^

The previous study has shown that elevation of inflammatory serum biomarkers is associated with the onset and recurrence of AF.^27^ Therefore, the role of inflammation in AF recurrence has been recently proposed as a new hypothesis to explain the high rate of occurrence and recurrence of AF.^11^ The study aims to find a potential biomarker to aid in risk stratification of AF patients after CBA.

hs‐CRP is a common inflammatory factor and is elevated following cardiomyocyte necrosis in patients with an AMI, having a prognostic value in the prediction of AF recurrence.^28^, ^29^ In our study, no significant difference was noted between the AF recurrence and AF nonrecurrence groups for the hs‐CRPpre and hs‐CRPpost levels, which may be because hs‐CRP is a nonspecific acute‐phase reactant synthesized by hepatocytes so that easily affected by other comorbidities.^30^

HMGB1 is a secreted protein that can be released into the extracellular space by damaged cells during injury. In addition, HMGB1 could be an inflammatory mediator that participates in the pathogenesis of inflammatory diseases.^13^ After CA, HMGB1 expression increases, causing a continuous state of oxidative stress and inflammation process.^31^ Furthermore, HMGB1 may bind to the HMGB1 receptor (Toll‐like receptor‐2,4, TLR‐2,4) located in the left atrium and mediate atrial remodeling.^15^ A study found TLR‐4 expression was an independent predictor of AF recurrence following cryoballoon‐based PV isolation for pAF.^32^ Therefore, exploring HMGB1 whether could be a potential predictor that can not only reflect the inflammatory level but is also associated with the myocardial injury level is of great significance for AF management.

To determine the impact of postoperative HMGB1 on AF recurrence, we analyzed 1‐year AF‐free survival in pAF patients after CBA. In contrast to previous studies, we collected blood samples from the left atrium instead of peripheral vessels to verify the effect of HMGB1. CA damages the myocardium and hence, HMGB1 is released by the injured cardiomyocytes directly into the left atrium. The collection of blood samples from the left atrium was aimed to emphasize the influence of local inflammation caused by HMGB1 on AF recurrence. Interestingly, in pAF patients with or without recurrence, the preoperative levels of HMGB1 were the same, but the levels of postoperative HMGB1 were significantly different between the groups. And HMGB1post level was associated with AF recurrence in pAF patients who underwent CBA.

Under stress, HMGB1 shuttles from the nuclear compartment to the cytoplasm, which indicates an elevation of intracellular HMGB1 levels.^33^ Therefore, the intracellular level of HMGB1 may be high despite the preoperative HMGB1 level in the left atrium showing no difference between AF recurrence and nonrecurrence. CBA allows the passive secretion of intracellular HMGB1, which results in an increase in HMGB1 in the left atrium as well as significant differences between recurrent and nonrecurrent AF. The exact mechanism needs to be verified by further cellular and animal experiments.

In conclusion, after CBA, HMGB1 is released into serum by damaged myocardial cells of the left atrium. In addition, HMGB1 acts as a proinflammatory factor in atrium remodeling, making atrial arrhythmia more likely to occur. This might explain the moderate predictive power of HMGB1post released from the left atrium for AF recurrence within 1 year.

Several studies have indicated that enlarged LA size is a potential predictor of AF recurrence with AF patients after CBA.^34^, ^35^ However, this conclusion is based on the study of patients with LA enlargement. In our small sample study, the mean LAVI was 33.42 ml/m^2^ and LVEF was 62.70%, indicating that our patients had only slight structural remodeling of LA. Thus, this conclusion is not suitable for our study.

## 5 LIMITATION

Our study had several limitations. First, in this study, the elevation of HMGB1 was after CBA, not before. Therefore, HMGB1post could aid in risk stratifying patients for AF recurrence, but they cannot be utilized as markers to assist ablation decisions. Second, our sample size was small and HMGB1 levels after ablation were adjusted for several available parameters. Therefore, the lack of adjustment for other important factors related to AF recurrence after ablation is another limitation of this study. We adopted the bootstrapping method (1000 repetitions) to compensate for the small sample size. And we intend to increase our sample size and follow up our patients for further study. Third, some AF events were asymptomatic and our patients were intermittently followed up depending on patient‐reported symptoms. Thus, the AF recurrence rates might have been underestimated, which can be improved by lengthening the follow‐up duration, more frequently, and using more long‐time monitoring tools, such as a loop recorder, or 7–14 days ECG patches.

## 6 CONCLUSION

Presently, there are no studies to explore the effect of local inflammation caused by CBA on AF recurrence, and no biomarkers that not only reflect inflammation but are also associated with myocardial injury to aid in risk stratifying patients for AF recurrence. Our study finds that in pAF patients undergoing CBA, HMGB1 released from the left atrium is associated with AF recurrence and has a moderate predictive power for AF recurrence within 1 year. Therefore, we offer a potential biomarker representing both myocardial injury and inflammation to assist in the risk stratification of AF patients after CBA. The identification of patients at risk for AF recurrence is of great significance as it may lead to prompt therapeutic interventions, thus improving prognosis and decreasing cardiovascular and cerebrovascular events.

## Supporting information

Supplementary information.

## Data Availability

Data are available on request due to privacy/ethical restrictions.
