# Supplementary material for: Predictive value of HMGB1 for atrial fibrillation recurrence after cryoballoon ablation in paroxysmal atrial fibrillation patients
Source: Clin Cardiol. 2022 Sep 20;45(12):1229–35. doi: 10.1002/clc.23904 (PMC9748753; doi:10.1002/clc.23904)
Supplement: Supplementary file 1 — Supplementary information. [file CLC-45-1229-s001.docx]

**SUPPLEMENTAL MATERIAL**

**Supplemental Figures and Figure Legend**


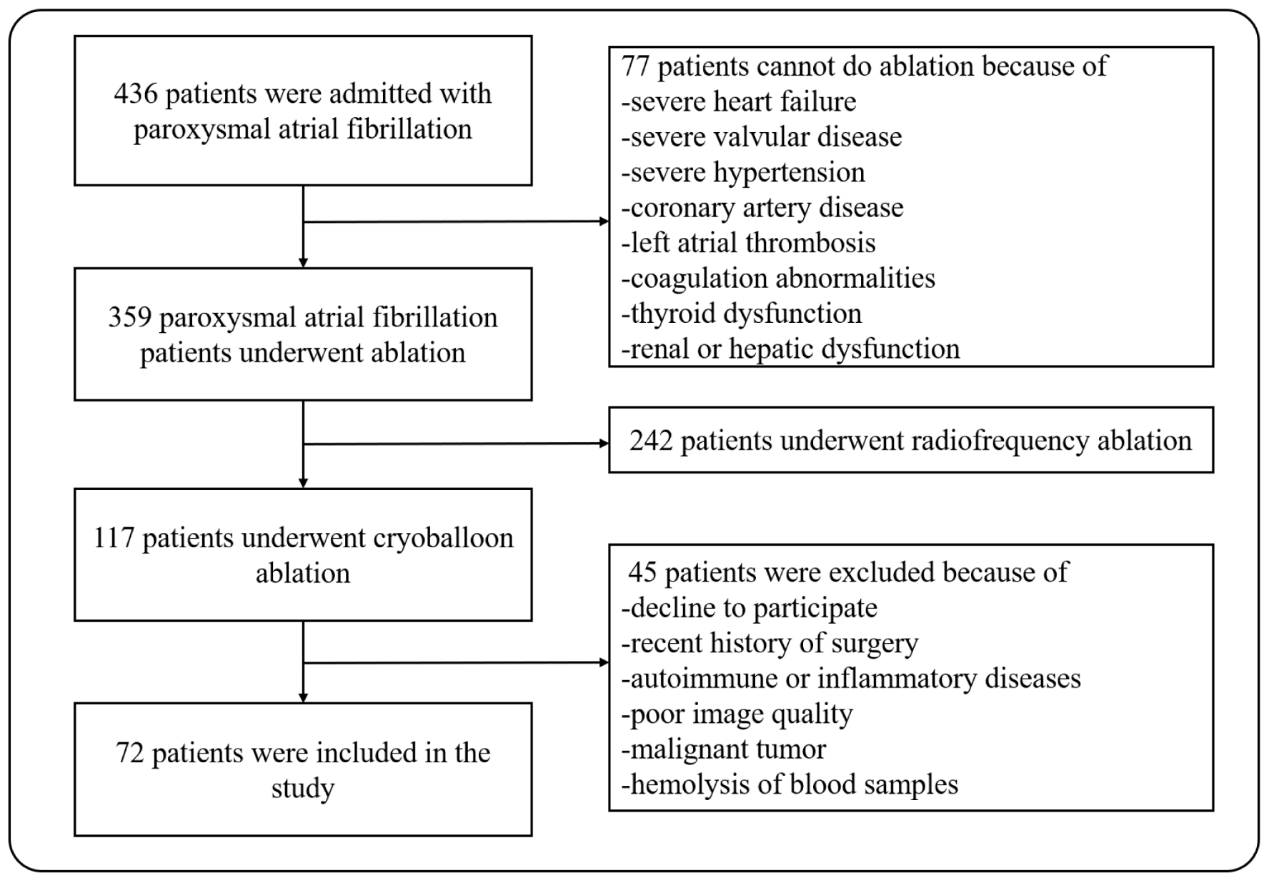


**Supplementary Figure S1. Patient recruitment flow chart.**

**Supplementary Table S1. Comparison of cryoballoon performance data between the two groups**

|  | All subjects | Nonrecurrence | Recurrence | P-value after boostrapping |
| --- | --- | --- | --- | --- |
|  | (N=72) | (N=53) | (N=19) |  |
| Nadir temperature (℃) |  |  |  |  |
| LSPV | -51.1±1.1 | -51.0±1.1 | -51.2±1.1 | 0.56 |
| LIPV | -51.1±1.2 | -50.9±1.2 | -51.5±1.3 | 0.10 |
| RSPV | -51.0±1.1 | -51.0±1.1 | -51.1±1.1 | 0.72 |
| RIPV | -51.1±1.1 | -51.0±1.2 | -51.2±1.0 | 0.50 |
| Application (n) |  |  |  |  |
| LSPV | 1.3±0.6 | 1.3±0.6 | 1.2±0.5 | 0.56 |
| LIPV | 1.3±0.5 | 1.3±0.5 | 1.4±0.6 | 0.54 |
| RSPV | 1.3±0.5 | 1.3±0.5 | 1.3±0.7 | 0.72 |
| RIPV | 1.3±0.5 | 1.3±0.6 | 1.2±0.4 | 0.38 |

The values shown are mean ± SD.

Abbreviation: LIPV, left inferior pulmonary vein; LSPV, left superior pulmonary vein; RIPV, right inferior pulmonary vein; RSPV, right superior pulmonary vein.
